# A Genetic Score of Predisposition to Low-Grade Inflammation Associated with Obesity May Contribute to Discern Population at Risk for Metabolic Syndrome

**DOI:** 10.3390/nu11020298

**Published:** 2019-01-30

**Authors:** Sebastià Galmés, Margalida Cifre, Andreu Palou, Paula Oliver, Francisca Serra

**Affiliations:** 1 NUO Group, Laboratory of Molecular Biology, Nutrition and Biotechnology, Universitat de les Illes Balears, 07122 Palma, Spain; s.galmes@uib.cat (S.G.); marga.cifre@uib.es (M.C.); Andreu.palou@uib.es (A.P.); francisca.serra@uib.es (F.S.); 2 CIBER de Fisiopatología de la Obesidad y Nutrición (CIBEROBN), 28029 Madrid, Spain; 3 Institut d’Investigació Sanitària Illes Balears (IdISBa), 07120 Palma, Spain

**Keywords:** genetic score, obesity, metabolic syndrome, low-grade inflammation, type 2 diabetes

## Abstract

Omega-3 rich diets have been shown to improve inflammatory status. However, in an ex vivo system of human blood cells, the efficacy of eicosapentaenoic acid (EPA) and docosahexaenoic acid (DHA) modulating lipid metabolism and cytokine response is attenuated in overweight subjects and shows high inter-individual variability. This suggests that obesity may be exerting a synergistic effect with genetic background disturbing the anti-inflammatory potential of omega-3 long-chain polyunsaturated fatty acids (PUFA). In the present work, a genetic score aiming to explore the risk associated to low grade inflammation and obesity (LGI-Ob) has been elaborated and assessed as a tool to contribute to discern population at risk for metabolic syndrome. Pro-inflammatory gene expression and cytokine production as a response to omega-3 were associated with LGI-Ob score; and lower anti-inflammatory effect of PUFA was observed in subjects with a high genetic score. Furthermore, overweight/obese individuals showed positive correlation of both plasma C-Reactive Protein and triglyceride/HDLc-index with LGI-Ob; and high LGI-Ob score was associated with greater hypertension (*p* = 0.047), Type 2 diabetes (*p* = 0.026), and metabolic risk (*p* = 0.021). The study shows that genetic variation can influence inflammation and omega-3 response, and that the LGI-Ob score could be a useful tool to classify subjects at inflammatory risk and more prone to suffer metabolic syndrome and associated metabolic disturbances.

## 1. Introduction

Obesity is currently considered one of the main health problems worldwide [1], and it is usually accompanied by the abnormal production of cytokines and adipokines by the immune system and adipose tissue, respectively, as well as by a disturbed profile of lipid mediators [2]. Although activation of inflammatory pathways is indispensable for the organism in specific circumstances, such as fighting against infections, undamaged inflammatory pathways are critical for tissue health and for proper homeostasis [3], including adipose tissue expansion and remodeling [4]. However, at some step that is still not well characterized, obesity results in a state of low-grade chronic metabolic inflammation involving many organs, altering energy and substrate fluxes and giving rise to the development of metabolic pathologies [5,6,7,8,9,10] (diabetes, dyslipidemia, and hypertension, among others) (see [3] for a comprehensive overview). According to the latest Obesity Update report of the Organization for Economic Co-operation and Development, at present more than one in two adults and nearly one in six children are overweight (OW) or obese (OB). The obesity epidemic continues its widespread rise and new projections until 2030 are keeping the same trend [11]. This discouraging landscape shows the need for more focused strategies that go far beyond the control of energy intake or expenditure, which may fit some people, but not all. At this respect, personalized or precision interventions for disease and health-risk mitigation are a very dynamic area of research [12]. This may drive to nutrition-based personalized interventions, aiming either to prevent the onset of obesity or to counteract its development in a more specific way. 

Meanwhile, the beneficial effects of food bioactive compounds, such as omega-3 long-chain polyunsaturated fatty acids (PUFA), mainly eicosapentaenoic (EPA) and docosahexaenoic acids (DHA), have been described in various studies. The cardio-protective potential of PUFA derives from their capacity to decrease circulating triglycerides, to show antihypertensive activity, to protect against the formation of thrombi, and to reduce inflammatory levels [13]. Furthermore, concerning low-grade inflammation associated with obesity, EPA and DHA are particularly interesting because they can be endogenously metabolized to pro-resolving lipid mediators which act as facilitators of timely resolution of inflammation [14,15,16,17]. In addition, the beneficial effects derived from PUFA intake may depend on factors, such as adiposity or genetics [18]. A number of studies have addressed the relationship between consumption of PUFA-rich foods and the presence of genetic variants influencing blood lipid profile, including high-density lipoprotein cholesterol (HDLc) [19,20,21], inflammation [22] or coronary artery disease risk [23]. In the present study, we aimed to assess whether genetic background could modulate inflammatory status, and influence the response associated to PUFA exposure in an in vitro system of human peripheral blood mononuclear cells (PBMC), a blood cell fraction composed mainly by lymphocytes and monocytes [24] bearing a transcriptome, which in vitro had been previously shown to reflect metabolic adaptations of key tissues involved in energy and lipid metabolism [25,26].

## 2. Materials and Methods

### 2.1. Study Participants and Design

**Figure 1:**
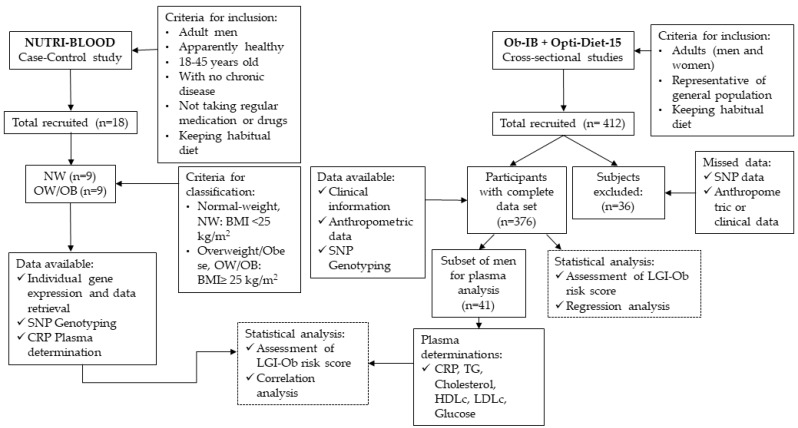
Flowchart diagram showing the main characteristics of the genetic association study to build up the score of predisposition to low-grade inflammation associated with obesity (LGI-Ob). This is based on three observational studies: One case-control (NUTRI-BLOOD), and two cross-sectional (OptiDiet-15 and Ob-IB) studies. NW (Normo-weight); OW/OB (Overweight/Obese); SNP (Single Nucleotide Polymorphism); CRP (C-reactive protein); BMI (Body Mass Index); TG (Triglyceride); HDLc (Cholesterol-High Density Lipoprotein); LDLc (Cholesterol-Low Density Lipoprotein).

This genetic association study is based on three observational studies: One case-control (NUTRI-BLOOD) and two cross-sectional (OptiDiet-15 and Ob-IB) studies (Figure 1). STREGA reporting guidelines were used. 

Initial samples were genotyped from participants in the NUTRI-BLOOD study (IB 2112/13 PI), in which 18 young apparently healthy males (aged from 19–36) were recruited (from October 2013 to November 2014) based on their body mass index (half of them normo-weight and the rest overweight or obese) to test the effect of DHA and EPA in isolated PBMC maintained in vitro [24]. Sample size was based on the number of subjects analyzed in similar experiments conducted in ex vivo PBMC, where the conditions were highly controlled [27,28]. Participant characteristics and data on the efficacy of the bioactive compounds have been previously published [24]. Results suggested that genetic background could influence the anti-inflammatory response observed in PBMC under omega-3 PUFA exposure, evaluated in the present study. Therefore, culture media and plasma of the participants were used for cytokine and C-reactive protein (CRP) determinations, respectively. Saliva samples were obtained for the present study and used for genotyping. Furthermore, individual records and data, including those concerning the response to PUFA treatments, were retrieved. 

In addition, in the present study, in order to increase the number of subjects and to prevent a potential source of bias, participants from the running studies OptiDiet-15 and Ob-IB were incorporated to perform the genotype analysis. The respective protocols for recruitment, procedures, and sampling were approved by the ethics committee (*Comitè d’Ètica de la Investigació de les Illes Balears, CEI-IB*). All participants gave written informed consent. Procedures were followed in accordance with the principles of the Declaration of Helsinki and all volunteer data were codified to guarantee anonymity accuracy. 

The OptiDiet-15 (IB 2569/15 PI) and Ob-IB (IB 2009/13) studies, both contributed to the present work by making available a representative sample of the general population of Mallorca (Spain). Participants were mostly Caucasian and from European origin (86% European, 11% South-American; and 3% others). Records on anthropometric, lifestyle, clinical and genetic data were collected. Individuals were recruited from September 2013 to May 2017. Only individuals from the OptiDiet-15 and Ob-IB studies with both complete clinical and genetic data sets were included in the final report (*n* = 376). To focus on the hypothesis, a representative subset of male individuals was further recalled according to their genetic and anthropometric characteristics from either OptiDiet-15 or Ob-IB, around 10% fulfilled the requirements (41 subjects) and were further analyzed as described below.

### 2.2. Anthropometric Measurements

Height, weight, body fat percentage (BF%), waist, and hip circumferences were measured with a non-elastic tape during face-to-face interviews by a trained investigator with the volunteer wearing minimum clothing. The waist-to-height ratio (WtHR) was calculated. The percentage of body fat was measured with a bio-impedance apparatus (OMRON BF306, Hoofddorp, The Netherlands). Body mass index (BMI) was calculated by the ratio between weight and the square of the height. Systolic (SBP) and diastolic blood pressure (DBP) were measured in triplicate with a sphygmomanometer (Omron HEM705CP, Hoofddorp, The Netherlands), measurement was made after the volunteer remained seated for ten minutes. Mean of arterial pressure (MAP) was calculated by the following formula: 2/3.DBP + 1/3.SBP [29]. The waist and hip data were only available for the OptiDiet-15 and Ob-IB studies.

### 2.3. Isolation, Culture Conditions and Gene Expression Determination in PBMC

Blood from NUTRI_BLOOD volunteers was collected using Vacutainer^®^ EDTA tubes and PBMC were isolated using Ficoll-Paque Plus density gradient media (GE Healthcare Bio Science, Madrid, Spain). Cells were incubated with RPMI-1640 medium (Sigma-Aldrich, St Louis, MO, USA) supplemented with 10% of foetal bovine serum, 1% of L-glutamine (2 mM) and a combination of 100 units/mL and 100 µg/mL of penicillin and streptomycin, respectively (Sigma-Aldrich, St Louis, United States). Incubated cells were treated with omega-3 PUFA (DHA and EPA), prepared as a 10 mM stock solution dissolved in ethanol, and administered to PBMC in a final concentration of 10 µM. Both omega-3 PUFA (>98% purity) were purchased from Sigma-Aldrich (St. Louis, MO, USA). This concentration was chosen based on our previous optimization experiments showing a maximum effect on gene expression. Control cells were incubated with ethanol.

After a 48 h-incubation period, total RNA was isolated using Direct-zol RNA Mini-Prep (Zymo Research Corp, Irvine, CA, USA). Equal amounts of total RNA (50 ng) were transcribed into cDNA using an iScrip cDNA synthesis kit (Bio-Rad Laboratories, Madrid, Spain) in an Applied Biosystems 2720 Thermal Cycler. Real-time Polymerase chain reaction (PCR) was performed for each Reverse transcription (RT) product to determine mRNA expression of genes using Power SYBR Green PCR Master Mix (Applied Biosystems, Madrid, Spain). All primers were purchased from Sigma Genosys (Sigma Aldrich Química SA, Madrid, Spain). Gene expression data were normalized against the housekeeping gene ribosomal protein, large, P0 (RPLP0).

Differences in mRNA expression of IL-6 and TNFα by isolated PBMC incubated with DHA and EPA were calculated in comparison with the respective untreated control cells. Summing up these values, an overall response in the expression of pro-inflammatory genes (ΔEPG) was obtained. Therefore, ΔEPG is an integrative value that reflects the feasibility of DHA and EPA in modulating the inflammatory potential; and a more negative ΔEPG would be suggestive of better responsiveness to the anti-inflammatory potential of the tested omega-3 PUFA in the respective individuals. 

### 2.4. Quantification of Circulating Parameters

Determination of glucose, total cholesterol (TC) and triglycerides (TG) in blood were assessed with an Accu-trend device (Roche Diagnostics, Barcelona, Spain). High-density lipoprotein cholesterol (HDLc) levels were measured in EDTA plasma by means of a colorimetric kit (Química Clínica Aplicada S.A., Tarragona, Spain) following the manufacturer’s instructions. Low-density lipoprotein cholesterol (LDLc) levels were calculated from Freideweld formula [30]: LDLc = TC − (HDLc + TG/5). C-reactive protein was measured with a commercial ELISA kit of Invitrogen (Thermo-Fisher Scientific, Waltham, MA, USA). 

### 2.5. DNA Isolation and Genotyping

Genomic DNA was isolated from volunteer saliva sample (400 µL) using High Pure PCR template Preparation Kit (Roche, Basel, Switzerland). The quality and the concentration of the isolated DNA were assessed using Nanodrop 1000 UV-Vis Spectrophotometer (Thermo-Fisher Scientific, MA, USA). A final concentration between 20–100 ng/µL of the isolated DNA was considered great. Then, aliquots were stored at −20 °C until the analysis of polymorphisms. Genotyping was performed by qPCR (LightCycler^®^480 *FastStart* DNA or Genotyping Master mix, Roche, Basel, Switzerland) using the differential melting temperature to determine the alleles of each single nucleotide polymorphism (SNP). The qPCRs were done following these conditions: 95 °C for 5 min, 45 cycles of denaturation at 92 °C for 15 s and annealing 60 °C for 1 min. The melting temperatures were recorded and analysed to determine the presence of specific alleles. The genotyping of all SNP was done using expressly designed probes (TIB Molbiol, Berlin, Germany). 

### 2.6. Elaboration of the Genetic Score of Predisposition to Low-Grade Inflammation Associated with Obesity (LGI-Ob)

Concerning the selection of genetic variants, we focused our interest on SNP related to an altered inflammatory profile, conferring major predisposition to obesity or higher cardiovascular risk. Genome-wide association studies (GWAS), meta-analyses and genetic association studies were the primary source for selection of the genes of interest. Additionally, to be part of the genetic score we imposed that the selected SNPs fulfilled the condition of being potentially modulated by lifestyle interventions, mainly by dietary factors. Therefore, preference was focused on SNP characterized by genotype-nutrient-phenotype associations, with the potential to modulate the genetic risk by external factors, and particularly in those nutrigenetic relationships involving polyunsaturated long chain fatty acids (Table 1). LGI-Ob genetic score is based on five SNP, a number that can be easily affordable in routinely analytical tests, each one associated with a representative gene of obesity-related metabolic disturbances and all of them known for their interaction with plasma inflammatory biomarkers. 

The main characteristics of the selected genes and SNP are summarized below. Two polymorphisms are located in the promoter and influence both gene expression and protein levels. Rs1800629 entails the substitution of a G for an A in the promoter of tumor necrosis factor alpha (*TNFα*) gene and increases both the transcription and production rates of the cytokine. A nutrigenetic interaction with omega 3 PUFA supplementation in the modulation of plasma CRP has been observed [31]; and associated with insulin resistance, higher levels of inflammatory metabolites in plasma [32], and other age-related alterations [33]. Rs5082, located in the promoter region of apolipoprotein A2 (*APOA2*) gene, has been shown to interact with dietary fatty acid intake to modulate inflammation [34,35], and is associated with both lower transcription rate and lower concentration of Apolipoprotein A-II in plasma [36], conferring higher risk of obesity and diabetes mellitus [37,38]. Furthermore, non-T subjects with high omega 3 PUFA intake show greater capacity for oxidative stress neutralization, whereas T carriers have been associated with resistance to increase superoxide dismutase 2 (SOD2) activity with omega 3 PUFA intake [39] and also with an increase in plasma oxidative biomarkers when they present a rich omega 6 PUFA diet [39].

The polymorphism rs4880 on SOD2 is located in the coding region and define a missense variant, it involves the change of Alanine to Valine at position 16 of the mitochondrial SOD2, which reduces free radical scavenging efficiency in the cell [40,41] and has been associated with altered plasmatic cytokine profile [42], with major risk to cardiovascular disease [43] type 2 diabetes [44], DNA damage [45] and micro- and macro-vascular complications in diabetes [46]. Interestingly, reduced cytokine production induced by the bioactive compound resveratrol in cultured PBMCs has been shown to be associated with the presence of this polymorphism, being less evident in Valine carriers [42]. Besides, Val risk allele carriers have been associated with lower benefits on inflammatory and lipid profile after lipid-lowering drug administration [47], but this genotype has also been associated with a lower risk of breast cancer in women who usually consume fish oil [48]. The last two SNP performing LGI-Ob genetic score have been associated with metabolic disturbances in different GWAs and other association studies. First, rs1260326 located in glucokinase regulator (GCKR) gene involves the change of the amino acid Proline at position 466 for Leucine and has been strongly associated with the risk of elevated CRP [49], dyslipidemia [49,50,51,52] and type 2 diabetes [53] in different GWAs. Nevertheless, two additional studies pointed out a potential modulation of omega 3 PUFAs on lipid profile and inflammatory plasma biomarkers [54,55]. Finally, we included the FTO gene, which codifies an alpha-ketoglutarate dependent dioxygenase and is one of the genes most strongly related with metabolic syndrome and obesity traits [56,57] and risk for type 2 diabetes (T2D) [58]. The intronic variant (rs9939609) has been recently analyzed by its influence on inflammatory biomarkers [56,58]. Despite the greater predisposition to obesity of AA subjects, the risk could be increased in the case of a PUFA:SFA ratio intake lower than 0.43 [59] or depending on the quality of dietary fat [59,60,61].

### 2.7. Assessment of the Metabolic Profile and Health Status

Concerning participants from the studies OptiDiet-15 and Ob-IB, information about suffering hypertension (HT), dyslipidemia (DL), T2D, or visceral obesity (VO) was collected to assess the prevalence of metabolic syndrome. Participants were considered at risk of HT when they met any of the following WHO criteria [69]: Systolic blood pressure (SBP) ≥130 mmHg, diastolic blood pressure (DBP) ≥80 mmHg, taking medication for HT, or with a medical diagnosis of HT. The individuals who reported diagnosis for DL and T2D were considered at risk for those conditions. VO was inferred from the WtHR; those individuals with WtHR ≥0.51 (women) or WtHR ≥0.53 (men) were considered at risk. Finally, participants with two or more alterations of those named above were considered as individuals at high metabolic risk (MR) (Table 2).

### 2.8. Statistical Analysis

Data analysis was performed with SPSS v21 (SPSS, Chicago, IL, United States). Normal distribution of variables was tested by the Shapiro-Wilk test when the number of cases was under 50 and by Kolmogorov-Smirnov test when the number of values was greater than 50. Homoscedasticity was tested using the Levene test; when variables did not show homoscedasticity criteria, they were either log transformed, or a non-parametric test was used. 

Population was stratified by BMI group (normal-weight, NW: BMI < 25 kg/m^2^ vs. overweight, OW/OB: BMI ≥ 25 kg/m^2^) or by genetic risk score (low score: <5 vs. high score: ≥5; based on the median value of the frequency distribution in general population (see point 3.3)). Subject characteristics, mainly to assess differences between NW and OW/OB groups, were assessed using Student’s *t* test. A Student’s paired *t*-test was used to compare levels of cytokine production between EPA or DHA treated cells and control (no-treated) cells within high or low score groups.

Assessment of the relationship between LGI-Ob genetic score (as a continuous variable) and biomarkers (plasma parameters and gene expression data) was assessed by Pearson’s or Spearman’s regression tests. After stratification by BMI groups, correlation analyses adjusted by age were computed between plasma parameters and the genetic score. The coefficient of regression between LGI-Ob genetic score and the number of metabolic alterations was obtained using linear regression adjusting by confounding variables. 

The contribution of the LGI-Ob genetic score (as a dichotomous variable) to find out the odds ratios (OR) for metabolic disorders or for metabolic risk were obtained by logistic regression, using low score subjects (<5 risk alleles) as reference (OR:1) and adjusting by the confounding variables age, gender, overweight (except for the variable VO), and ethnicity. Other potential confounding factors were non-reported or unknown.

Receiver operating characteristics (ROC) curve analysis was applied to test the diagnostic ability of LGI-Ob genetic score to classify potential individuals for obesity associated metabolic disturbances. No comparisons involving multiple simultaneous statistical tests were performed. The threshold of significance was defined at *p* < 0.05 for all analyses.

## 3. Results

### 3.1. Anti-inflammatory Potential of EPA and DHA is Influenced by LGI-Ob Genetic Score

**Figure 2:**
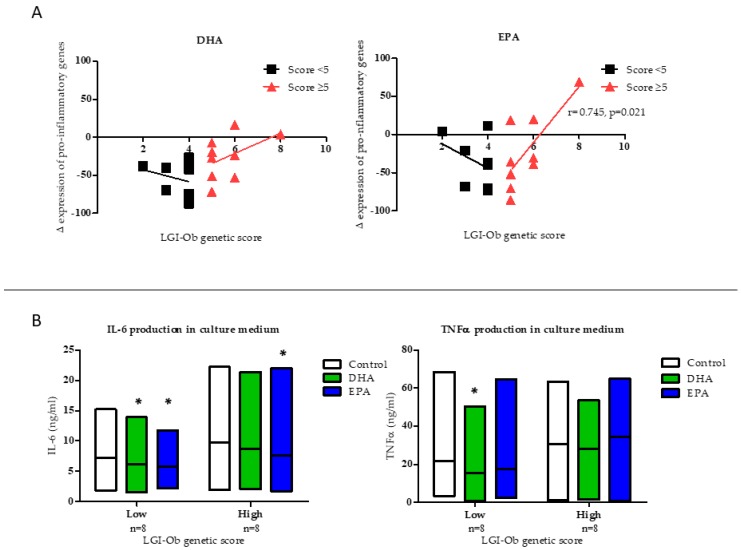
Effects of Docosahexaenoic Acid (DHA, 10 µM) and Eicosapentaenoic Acid (EPA, 10 µM) on (**A**) overall response in the expression of pro-inflammatory genes (ΔEPG), in PBMC measured by Reverse Transcription quantitative Polymerase Chain Reaction (RT-qPCR), and represented according to the LGI-Ob genetic score of the individuals. Cells were incubated for 48h with DHA and EPA and the effects in each individual were calculated in comparison with the respective untreated control cells. Pearson correlations were used to assess linear association between LGI-Ob genetic score and ΔEPG; (**B**) IL-6 and TNFα production in PBMC culture media by LGI-Ob genetic score: Low (<5) or high (≥5). Two-way repeated measures ANOVA was performed to compare differential cytokine production from control cells vs EPA or DHA treated cells in both LGI-Ob genetic score groups (* *p* < 0.05). Boxes represent the dispersion (maximum and minimum) and the horizontal line represents the arithmetic mean in each treatment. Data correspond to subjects from NUTRI-BLOOD study (*n* = 18).

Analysis of gene expression focusing on the feasibility of omega-3 PUFA to modulate inflammatory response (ΔEPG) and taking into account genotype characteristics of the individuals indicated that cellular response to DHA and EPA was dependent on the number of risk alleles carried. Thus, modulation of the expression of pro-inflammatory genes (ΔEPG) by treatment with the omega-3 fatty acids showed a different profile related to the LGI-Ob genetic score of the individuals (Figure 2A). ΔEPG correlated positively with the LGI-Ob score in subjects with a high score (≥5) and reached statistical significance in the case of treatment with EPA (*r* = 0.669, *p* = 0.049; Figure 2A). However, a negative, although not significant, correlation was found in subjects with a low score (<5), which was in agreement with decreased pro-inflammatory cytokines in the medium of the cells (PBMC) treated with the fatty acids (Figure 2B). In particular, both DHA and EPA were effective in decreasing the production of cytokines in culture media of PBMC from subjects with a low genetic score. Decreased IL6 was found after incubation with both DHA (Δ = −1.057, *p* = 0.035) and EPA (Δ = −1.450, *p* = 0.036) and decreased TNFα concentration was observed after DHA treatment (Δ = −6.175, *p* = 0.043) in samples from individuals with low score. However, in subjects with a high score only EPA decreased IL6 concentration in the media (Δ = −2.041, *p* = 0.042) (Figure 2B). Thus, these data show that a previous heterogeneous inter-individual response to omega-3 PUFA observed in an in vitro system of PBMC of the characterized subjects [24] could be related to the presence of genetic variants linked with a status of low-grade inflammation associated with obesity. The selected genes and role of the variants included in the LGI-Ob score shaped the anti-inflammatory response to PUFA, showing that individuals with elevated score were to some extent resistant to the anti-inflammatory potential of EPA and DHA, an effect observed at the level of both transcriptional and translational processes. 

### 3.2. Plasma Biomarkers of Inflammation are Influenced by LGI-Ob Genetic Score

**Figure 3:**
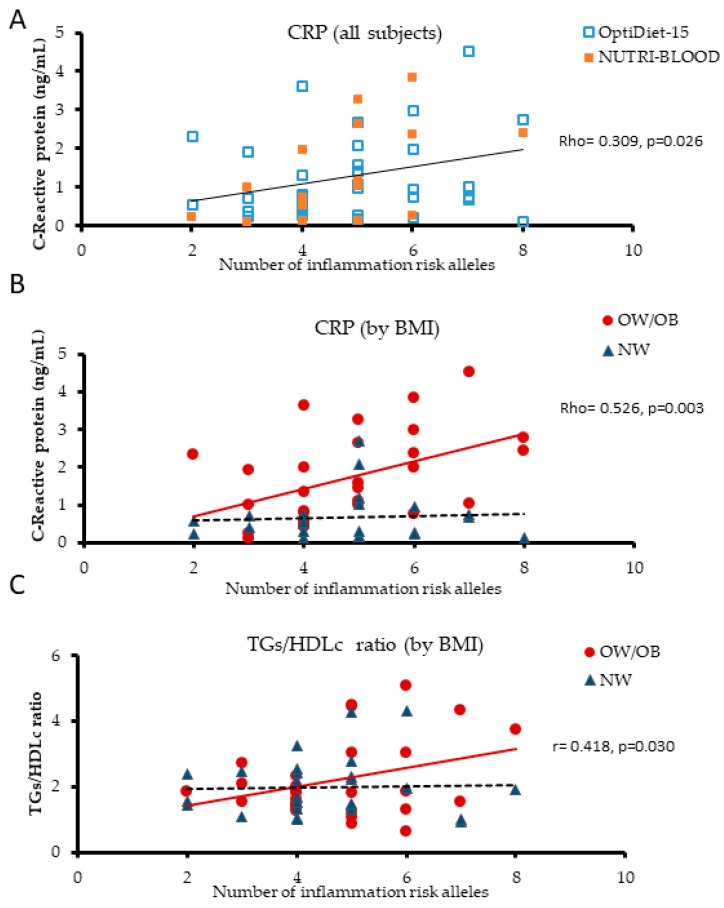
Linear correlation between plasma biomarkers and LGI-Ob genetic score. Relationship between C-reactive protein (CRP) and LGI-Ob genetic score in subjects classified by (**A**) source of study and (**B**) BMI: Normoweight (NW, BMI < 25); overweight/obese (OW/OB, BMI ≥ 25) (*n* = 52). (**C**) Relationship between triglycerides-to-HDLc (TGs/HDLc) ratio and LGI-Ob genetic score in subjects grouped by BMI (*n* = 55). Spearman and Pearson (adjusted by age) correlation analysis were applied. Rho: Spearman’s correlation coefficient; *r*: Pearson’s correlation coefficient. Data belong to both NUTRI-BLOOD (*n* = 18) and OptiDiet-15 (*n* = 41) studies.

To further assess the extent of genetic impact on inflammation, CRP levels were measured in plasma from the above individuals (volunteers of the NUTRI-BLOOD study, *n* = 18). A positive correlation (Rho = 0.580 *p* = 0.015) was observed between CRP concentration and the LGI-Ob score. Interestingly, stratification by BMI gave a stronger correlation when only OW/OB (BMI ≥ 25) individuals were included (Rho = 0.737, *p* = 0.023, *n* = 9), which was lost for NW subjects (BMI < 25) (Rho = 0.275, *p* = 0.509, *n* = 8). To rule out these data as a result of the specific population studied or associated with the low number of individuals analysed, data from the initial core group of participants (NUTRI-BLOOD study, *n* = 18) were pooled with a subset of individuals from Optidiet-15 study (*n* = 41), all of them adult men and representative of the general population (Table 3). Accordingly, subjects covered a wider range of age (19–74 years old) and overweight and obesity were more prevalent in individuals 10 years older than in normo-weight subjects (*p* = 0.002). After grouping by BMI, higher MAP (*p* = 0.037), plasma triglycerides (*p* = 0.016), and CRP (*p* < 0.0001) levels were observed in OW/OB subjects, but no differences were found in glucose, or cholesterol plasma levels (Table 3). Positive correlations were found between CRP and obesity related indicators, such as BMI (Rho = 0.664, *p* < 0.0001), body weight (Rho = 0.554, *p* < 0.0001), and %BF (Rho = 0.612, *p* < 0.0001; *n* = 52). Correlation analyses confirmed the positive relationship between plasma CRP and LGI-Ob genetic score (Rho = 0.309, *p* = 0.026; *n* = 52) (Figure 3A), as well as the positive correlation found in OW/OB subjects (Rho = 0.526, *p* = 0.003; *n* = 29), and the lack of it in NW individuals (Rho = 0.027, *p* = 0.903; *n* = 23). Analyses performed after adjusting by age, a potential confounder co-variable, remained significant (*r* = 0.503, *p* = 0.005) in the overweight/obese group, but not in NW group (*r* = 0.056, *p* = 0.806) (Figure 3B). In addition, we tested the association between CRP in the non-segregated population using the simple linear regression model adjusted by age and by BMI (*p* = 0.059) and adjusted by age and presence of overweight (*p* = 0.018). Therefore, the risk of higher basal inflammation entailed by adiposity seems to affect individuals unequally depending on their LGI-Ob genetic score. Furthermore, NW subjects with a high genetic score would be more susceptible to low-grade chronic inflammation in case of increasing body fat. 

Elevated levels of TG and low levels of HDLc have been described as key contributors to the development of inflammation and atherosclerosis [70], being associated with insulin resistance and T2D [71,72,73,74]. Although HDLc was not altered in the obese population studied, TG levels were 36% higher than in NW subjects (Table 3). Therefore, a positive correlation between the TG/HDLc ratio and LGI-Ob score was also found in overweight/obesity group (*r* = 0.418, *p* = 0.030; *n* = 28), adjusting by age, but not among NW subjects (Figure 3C). Using the same model (adjusted by age and overweight), the association between TG/HDL ratio and LGI-Ob score remained significant (*p* = 0.038); and if adjusted by BMI, the significance was not reached either (*p* = 0.061). Then, to determine the potential interaction between BMI and the genetic risk score, in relation to the levels of CRP and TG/HDL, both variables were dichotomized, and a two-way ANOVA test was performed, adjusting by age. Under these conditions, simple effects were statistically significant, but interaction effect was not seen.

Recently, the TG/HDLc ratio has been proposed as a reliable biochemical index predictor for insulin resistance, particularly in overweight diabetic patients [75]. Our results allow for a further level of precision, suggesting that the presence of the risk alleles considered in LGI-Ob genetic score is able to influence the TG/HDLc ratio. Therefore, individuals with a high genetic score are more susceptible to showing a disturbed ratio, and would thereby be at higher risk to develop insulin resistance and associated co-morbidities [71]. 

### 3.3. LGI-Ob Genetic Score May Contribute to Identify Subjects at Higher Risk to Develop Metabolic Syndrome

**Figure 4:**
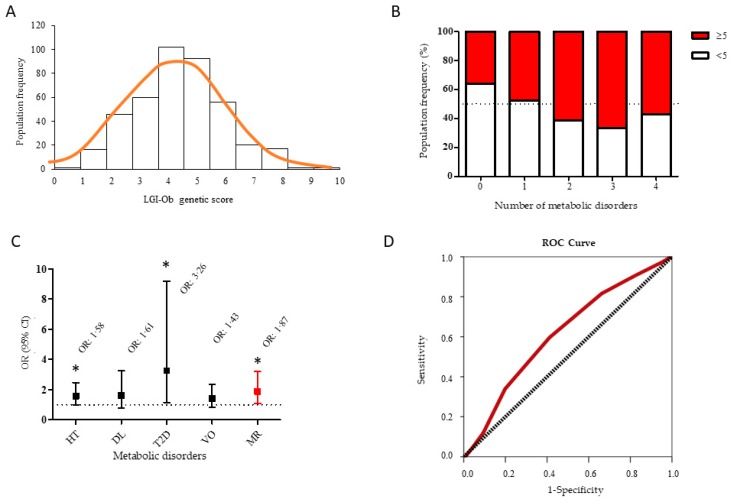
(**A**) Distribution of the frequency of LGI-Ob genetic score computed in the general adult population studied (*n* = 376); (**B**) Prevalence of the number of metabolic disorders suffered by the population analysed and categorised according to low (<5, *n* = 201) or high (≥5; *n* = 175) LGI-Ob genetic score; (**C**) Odds ratio (OR) for the metabolic disorders: Hypertension (HT), dyslipidemia (DL), type 2 diabetes (T2D), visceral obesity (VO), and metabolic risk (MR) of individuals with high LGI-Ob score (≥5) in comparison with individuals with low LGI-Ob score (<5). OR was estimated by binary logistic regression taking as a reference (OR = 1, indicated by the dashed line) individuals with low LGI-Ob score and adjusting for age, sex, and ethnicity. (**D**) Receiver operating characteristics (ROC) curve analysis showing the performance of LGI-Ob genetic score to classify potential individuals for obesity-associated metabolic disturbances.

All the above data point to the fact that the LGI-Ob genetic score could be relevant in mediating a higher rate of adverse events in the relationship with obesity and cardio-metabolic health. The next step was to assess the association of genetic score with known risk factors of metabolic syndrome in a wider, representative sample of the general adult population, incorporating the female gender. Information about health status and genotypes of interest was obtained from 376 subjects (63% women) (Table 4). The frequency of LGI-Ob score followed a normal distribution with a median of 4 (IQR = 2) and a mean of 4.36 (SD = 1.7) alleles of risk (Figure 4A). Therefore, the median value was used to stratify the subjects in two subgroups of genetic risk: Low (<5) and high score (≥5) throughout the study. 

Additionally, information concerning the presence of metabolic alterations (i.e., hypertension, dyslipidaemia, diabetes, and visceral obesity) in these subjects was recorded and analysed. Interestingly, the number of altered metabolic alterations showed a positive linear regression with the LGI-Ob genetic score (*β* = 0.145, *p* = 0.001). In fact, higher prevalence of two or more metabolic alterations was observed in individuals with a high genetic score, whereas individuals with a low score were more represented in the categories with no or only one metabolic alteration (Figure 4B). In order to characterise the prevalence of metabolic risk, a logistic regression analysis was undertaken. When compared to low score subjects (OR = 1), the high scoring group had higher odds ratio for all metabolic alterations registered, and reached statistical significance for hypertension (OR = 1.58, *p* = 0.047), T2D (OR = 3.26, *p* = 0.026), and for the presence of two or more risk factors (relative metabolic risk, MR) (OR = 1.87, *p* = 0.021) (Figure 4C). To check the accuracy of predicting the incidence of metabolic disturbances associated with obesity, MR variable was selected as a binary outcome and ROC analysis was performed. The ROC analysis showed a significant discriminant accuracy of the LGI-Ob in identifying subjects having metabolic disturbances related with obesity (Area Under Curve, AUC= 0.613, *p* = 0.001, confidence interval, CI = 0.551–0.674) (Figure 4D). Genetic variants in FTO gene are highly relevant in obesity and to assess whether LGI-Ob score would perform better than FTO on its own, we tested the removal of FTO polymorphism from the model. This had a minor impact on AUC value (AUC = 0.582, *p* = 0.036), suggesting that the remaining SNP in LGI-Ob model had major influence with the inflammatory profile associated with obesity than FTO *per se*. Therefore, the LGI-Ob score as initially defined (i.e., including the influence of FTO polymorphism) would support a higher degree of confidence in the discriminant accuracy of LGI-Ob score identifying subjects having metabolic disturbances related with obesity.

## 4. Discussion

Obesity is a multifactorial condition associated with metabolic disturbances, such as type 2 diabetes and cardiovascular diseases. Its treatment and prevention are difficult as it is highly influenced by both genetic and environmental factors, as well as by their interactions. In fact, the contribution of genetics to obesity and adiposity traits has been extensively analyzed and, to date, over 500 loci have been identified and constitute targets for active research (see [76,77] for recent reviews). Interestingly, functional characterization of the genes involved has revealed the role of immune-related cells and pathways, which pin-points the relevance of low-grade inflammation observed in obesity [3]. Thus, preventive or therapeutic strategies aiming to counteract immune-metabolic dysfunction may constitute an essential step in resolving obesity. In this respect, long chain omega 3 PUFA have been shown to induce an anti-inflammatory profile at the level of gene expression in immune cells [78,79,80]. In a previous study, we contributed to show the anti-inflammatory potential of EPA and DHA in humans, measuring the expression of inflammatory key genes and determining levels of cytokines in the medium of an in vitro system of PBMC isolated from apparently healthy adult men. However, the attenuated anti-inflammatory reaction was observed in cells from overweight/obese individuals, together with highly heterogeneous inter-individual responses [24]. Thus, in the present study we aimed to investigate to what extent inflammatory status and the beneficial effect associated with long chain omega 3 PUFA would be modulated by genetic background. A number of nutrigenetic studies have implemented the use of genetic risk scores, as they seem to have greater power than single variants and may be useful in elucidating the biological function of the SNP [77,81]. Therefore, a genetic score expected to identify the risk of Low-Grade Inflammation associated with Obesity (LGI-Ob) was elaborated. The score was based on the presence of 5 common variants known for their influence on plasma inflammatory biomarkers (TNFα and CRP), some of which have already been analyzed as regards PUFA interaction [31,34,42,53,56]. Application of LGI-Ob genetic score was initially performed in the core group of participants of the previous study, then it was tested in a medium-sized group of men, and finally in a totally heterogeneous sample of people representative of the general Caucasian population. The LGI-Ob genetic score followed a bell-shaped distribution with an observed range from 0 to 10. Taking the median value of the whole population, the score was predictive of higher risk (odds ratio) of metabolic disturbances, reaching statistical significance for HT, T2D, and MR. Furthermore, when the genetic predictor was contrasted with phenotypic features in the smaller subsets of individuals, it was positively correlated with a disturbed TG/HDL ratio, higher CRP, and also even decreased anti-inflammatory response to EPA and DHA. ROC analysis of the LGI-Ob score as a predictor of developing metabolic disturbances and associated low-grade inflammation gave an AUC_ROC_ of 0.613, which is not a very high value. However, the current genetic models to predict obesity based on SNP associated with BMI loci deal with AUC_ROC_ between 0.546 (when FTO is the only gene considered) up to 0.575 (when considering 12 or 32 loci) [82]. Therefore, available genetic information is relatively poor and does not allow for accurate discrimination between those at high risk of obesity. The LGI-Ob score identified in our study may represent a good genetic marker and may be useful at earlier steps aimed at evaluating an individual’s predisposition to metabolic disturbances associated with obesity and low-grade inflammation. Thus, an individual’s genetic information may enable the prevention and management of metabolic syndrome, following the current trend towards personalized nutritional approaches, which in addition may efficiently contribute to tailored dietary advice aiming for precision nutrition [12,83]. In summary, our results show that genetic data could be combined with other usual analytical parameters (i.e., CRP, HDLc, and TG) and/or contrasted with a functional test assessing in vitro performance of DHA/EPA concerning anti-inflammatory response. In this regard, the possibility of using the results from functional assays, for example testing in PBMC the response to omega 3, or other bioactive compounds with anti-inflammatory potential, can help determine the most appropriate intervention for each individual, as stated in previous works [24,84,85]. The knowledge provided by introducing the analysis of specific genetic variants could help to clarify some of the null or contradictory results found on Omega-3 supplementation studies [86,87].

Obesity may be exerting a synergistic effect with genetic background to disturb the anti-inflammatory potential of DHA and EPA. Hence, this study highlights that a set of relevant genetic variants associated with genes involved in energy metabolism and inflammatory processes could contribute to a better prediction of an individual’s metabolic resistance to the anti-inflammatory capacity of omega 3 PUFA. Therefore, the genetic score of predisposition to low-grade inflammation associated with obesity (LGI-Ob) defined in this study may efficiently contribute to discern population at risk for metabolic syndrome.

## Figures and Tables

**Table 1:** Genes and single nucleotide polymorphism (SNP) forming part of the LGI-Ob genetic score ^1^.

		Genotype			Associated Risk ^2^			Biomarkers/risk associated with risk allele	Interactions described concerning the risk allele
Rs code	Gene	a	b	c	a	b	c		
**rs1800629**	TNFα	GG	GA	AA	0	2	2	TNFα, CRP [31] CAD risk [62] IL18, IL18:IL-0 and TNFα:IL10 ratios [63] Ischemic Stroke [64] Disturbed plasma levels of PUFA [65]	Higher CRP levels after fish oil supplementation [31] Resistance to improve in inflammatory CRP plasmatic profile after dietary intervention [32]
**rs5082**	APOA2	CC	TC	TT	0	1	2	CRP [34,35] Obesity and Insulin Resistance [37,38]	More prone to high CRP levels in obesity [35] Protective effect against T2D, but not in case of obesity [35] Higher intakes of omega 3 are not associated with increases of SOD activity [39] Higher intakes of omega 6 are associated with increased oxidative biomarkers [39]
**rs4880**	SOD2	CC	CT	TT	0	1	2	IL6, TNFα, IL1β [42] CAD risk [43] T2D risk [44] DNA damage [45]	Attenuated response to statin drugs regarding lipid and inflammatory profile [47] Less risk of Breast Cancer in women when the consumption of fish or vegetables are high [48]
**rs1260326**	GCKR	CC	CT	TT	0	1	2	CRP [49,66] TAGs [49,50,51] TC [52] T2D risk [53]	Omega 3 PUFAs interaction regarding triglycerides levels [54] Lack of lowering effect of omega 3 PUFA on fasting insulin, HOMA-IR and CRP [55]
**rs9939609**	FTO	TT	TA	AA	0	1	2	BMI, BF [56] BMI [57] T2DM risk [67] CRP [56,58]	Lower PUFA:SFA ratio are associated with higher obesity risk than in TT subjects [59] Lower effect of diet/lifestyle interventions [68]

^1^ Genes and SNP included in the genetic score to estimate the predisposition to low-grade inflammation associated with obesity (LGI-Ob). Identification code (rs), associated gene, and risk value assigned to the respective genotype is shown together with related biomarkers and observed gene-diet or lifestyle interactions. ^2^ The weight indicated for each genotype has been obtained from published evidence. Abbreviations: APOA2 (apolipoprotein A2); BMI (body mass index); BF (body fat percentage); CAD (Cardiovascular disease); CRP (C-reactive protein); DHA (Docosahexaenoic acid); FTO (Fat mass and obesity-associated gene); GCKR (glucokinase regulator); HOMA-IR (Homeostatic Model Assessment of Insulin Resistance); IL (interleukin); PUFA (Polyunsaturated Fatty Acid); SFA (Saturated Fatty Acid); SOD2 (Superoxide dismutase 2); T2D (Type 2 Diabetes); TAGs (Triglycerides); TC (Total Cholesterol); TNFα (tumor necrosis factor alpha).

**Table 2:** Criteria used to assess the health status and risk of the subjects ^1^.

Metabolic Condition	No	Yes
**HT**	No diagnosis of HT and Not receiving antihypertensive treatment and SBP < 130 mmHg and DBP< 80 mmHg	Diagnosis of HT or Receiving antihypertensive treatment or SBP ≥ 130 mmHg or DBP ≥ 80 mmHg
**DL**	No diagnosis of DL and Not receiving lipid-lowering medication	Diagnosis of DL and/or Receiving lipid-lowering medication
**T2D**	No diagnosis of T2D and Not receiving antidiabetic treatment	Diagnosis of T2D or Receiving antidiabetic treatment
**VO**	WtHR < 0.51 (women) or < 0.53 (men)	WtHR ≥ 0.51 (women) or ≥ 0.53 (men)
**MR**	<2 of the previous conditions	≥2 of the previous conditions

^1^ Criteria used for the consideration of individuals at risk for different conditions related to metabolic disturbances. Abbreviations: DBP: Diastolic blood pressure; DL: Dyslipidemia; HT: Hypertension; MR: Metabolic Risk; SBP: Systolic blood pressure; T2D: Type 2 Diabetes; VO: Visceral obesity; WtHR: Waist-to-height ratio.

**Table 3:** Subjects characteristics ^1^.

	Subset Population (*n* = 59)		BMI < 25 (kg/m^2^) (*n* = 28)		BMI ≥ 25 (kg/m^2^) (*n* = 31)		
	Mean	SD	Mean	SD	Mean	SD	*p*-Value
Age (years)	33.4	13.8	28.0	9.6	38.3	15.2	0.002
**Anthropometric measures**							
Height (m)	1.80	0.07	1.80	0.07	1.80	0.07	0.696
Weight (kg)	81.7	18.2	68.8	8.7	93.4	16.5	<0.0001
BMI (kg/m^2^)	26.4	5.5	22.1	2.0	30.4	4.7	<0.0001
Body fat percentage (%)	23.6	7.7	18.1	5.7	28.6	5.6	<0.0001
**Cardiovascular health indicators**							
SBP (mmHg)	132.5	15.6	128.6	14.7	136.0	15.8	0.056
DBP (mmHg)	80.3	11.5	77.3	9.3	83.0	12.8	0.055
MAP	97.7	11.7	94.4	10.1	100.7	12.3	0.037
**Circulating parameters**							
Glucose (mg/dL)	96.10	28.35	91.93	25.08	100.00	31.01	0.195
Total cholesterol (mg/dL)	190.39	45.74	184.71	36.26	195.86	53.42	0.313
LDLc (mg/dL)	106.90	39.30	104.43	34.12	109.29	44.21	0.645
HDLc (mg/dL)	60.57	18.65	61.61	18.25	59.64	19.26	0.695
Triglycerides (mg/dL)	120.30	58.69	101.71	30.82	138.24	72.77	0.016
CRP (ng/mL)	1.26	1.10	0.67	0.63	1.73	1.18	<0.0001

^1^ Main characteristics of the subset of population characterised as a whole and grouped by BMI. This includes subjects from NUTRI-BLOOD study (*n* = 18) pooled with OptiDiet-15 (*n* = 41). The data are presented as the mean and standard deviation (SD). *p*-value was assessed by Student’s *t* test comparing BMI groups. Abbreviations: BMI: body mass index; CRP: C-reactive protein; DBP: diastolic blood pressure; HDLc: high density lipoprotein cholesterol; LDLc: low density lipoprotein cholesterol; MAP: mean of arterial pressure; SBP: systolic blood pressure.

**Table 4:** General population characteristics ^1^.

	General Adult Population	
	Mean	SD
**Demographic Descriptors**		
Gender (% female)	63.0	
Age (years)	36.0	15.0
European (%)	86.4	
Overweight/obese (%)	41.0	
**Anthropometric measures**		
Height (m)	1.67	0.90
Weight (kg)	69.7	16.3
Hips (cm)	95.6	11.2
Waist (cm)	84.1	15.8
Waist to Height Ratio	0.50	0.10
BMI (kg/m^2^)	24.9	5.2
Body fat percentage (%)	29.3	8.9
**Cardiovascular health indicators**		
SBP (mmHg)	125.4	15.6
DBP (mmHg)	73.1	9.9
MAP	72.1	33.1

^1^ Main characteristics of general adult population studied (*n* = 376). Only individuals with the whole set of data were considered. Data are presented as percentage or as the mean and standard deviation (SD). Overweight/obese: Subjects with BMI ≥ 25. BMI: body mass index; DBP: diastolic blood pressure; MAP: mean of arterial pressure; SBP: systolic blood pressure.

## References

[1] Saklayen M.G. (2018). The global epidemic of the metabolic syndrome. Curr. Hypertens. Rep..

[2] Ohman M.K., Shen Y., Obimba C.I., Wright A.P., Warnock M., Lawrence D.A., Eitzman D.T. (2008). Visceral adipose tissue inflammation accelerates atherosclerosis in apolipoprotein E-deficient mice. Circulation.

[3] Hotamisligil G.S. (2017). Inflammation, metaflammation and immunometabolic disorders. Nature.

[4] Wernstedt Asterholm I., Tao C., Morley T.S., Wang Q.A., Delgado-Lopez F., Wang Z.V., Scherer P.E. (2014). Adipocyte inflammation is essential for healthy adipose tissue expansion and remodeling. Cell Metab..

[5] Lumeng C.N., Saltiel A.R. (2011). Inflammatory links between obesity and metabolic disease. J. Clin. Investig..

[6] Cinti S., Mitchell G., Barbatelli G., Murano I., Ceresi E., Faloia E., Wang S., Fortier M., Greenberg A.S., Obin M.S. (2005). Adipocyte death defines macrophage localization and function in adipose tissue of obese mice and humans. J. Lipid Res..

[7] Blüher M. (2009). Adipose tissue dysfunction in obesity. Exp. Clin. Endocrinol. Diabetes.

[8] Donath M.Y., Shoelson S.E. (2011). Type 2 diabetes as an inflammatory disease. Nat. Rev. Immunol..

[9] Martinez B.K., White C.M. (2018). The emerging role of inflammation in cardiovascular disease. Ann. Pharmacother..

[10] Ochoa C.D., Wu R.F., Terada L.S. (2018). ROS signaling and ER stress in cardiovascular disease. Mol. Aspects Med..

[11] OECD Obesity Update 2017. www.oecd.org/health/obesity-update.htm.

[12] Schork N.J., Goetz L.H. (2017). Single-subject studies in translational nutrition research. Annu. Rev. Nutr..

[13] Allayee H., Roth N., Hodis H.N. (2009). Polyunsaturated fatty acids and cardiovascular disease: Implications for nutrigenetics. J. Nutrigenet. Nutrigenomics.

[14] Neuhofer A., Zeyda M., Mascher D., Itariu B.K., Murano I., Leitner L., Hochbrugger E.E., Fraisl P., Cinti S., Serhan C.N. (2013). Impaired local production of proresolving lipid mediators in obesity and 17-HDHA as a potential treatment for obesity-associated inflammation. Diabetes.

[15] Basil M.C., Levy B.D. (2016). Specialized pro-resolving mediators: Endogenous regulators of infection and inflammation. Nat. Rev. Immunol..

[16] Chilton F.H., Dutta R., Reynolds L.M., Sergeant S., Mathias R.A., Seeds M.C. (2017). Precision nutrition and omega-3 polyunsaturated fatty acids: A case for personalized supplementation approaches for the prevention and management of human diseases. Nutrients.

[17] Clària J., López-Vicario C., Rius B., Titos E. (2017). Pro-resolving actions of SPM in adipose tissue biology. Mol. Aspects Med..

[18] Jourdan C., Kloiber S., Nieters A., Seiler H., Himmerich H., Kohli M.A., Lucae S., Wolfram G., Gieger C., Wichmann H.-E. (2011). Gene-PUFA interactions and obesity risk. Br. J. Nutr..

[19] Bodhini D., Gaal S., Shatwan I., Ramya K., Ellahi B., Surendran S., Sudha V., Anjana M.R., Mohan V., Lovegrove J.A. (2017). Interaction between TCF7L2 polymorphism and dietary fat intake on high density lipoprotein cholesterol. PLoS ONE.

[20] Warodomwichit D., Arnett D.K., Kabagambe E.K., Tsai M.Y., Hixson J.E., Straka R.J., Province M., An P., Lai C.-Q., Borecki I. (2009). Polyunsaturated fatty acids modulate the effect of TCF7L2 gene variants on postprandial lipemia. J. Nutr..

[21] Lai C.-Q., Corella D., Demissie S., Cupples L.A., Adiconis X., Zhu Y., Parnell L.D., Tucker K.L., Ordovas J.M. (2006). Dietary intake of n-6 fatty acids modulates effect of apolipoprotein a5 gene on plasma fasting triglycerides, remnant lipoprotein concentrations, and lipoprotein particle size: The framingham heart study. Circulation.

[22] Noumi Y., Kawamura R., Tabara Y., Maruyama K., Takata Y., Nishida W., Okamoto A., Nishimiya T., Onuma H., Saito I. (2018). An inverse association between serum resistin levels and n-3 polyunsaturated fatty acids intake was strongest in the SNP-420 G/G genotype in the Japanese cohort: The toon genome study. Clin. Endocrinol..

[23] Martinelli N., Girelli D., Malerba G., Guarini P., Illig T., Trabetti E., Sandri M., Friso S., Pizzolo F., Schaeffer L. (2008). FADS genotypes and desaturase activity estimated by the ratio of arachidonic acid to linoleic acid are associated with inflammation and coronary artery disease. Am. J. Clin. Nutr..

[24] Cifre M., Díaz-Rua R., Varela Calviño R., Reynés B., Pericás-Beltrán J., Palou A., Oliver P. (2016). Human peripheral blood mononuclear cell in vitro system to test the efficacy of food bioactive compounds: Effects of polyunsaturated fatty acids and their relation with BMI. Mol. Nutr. Food Res..

[25] Reynés B., Díaz-Rúa R., Cifre M., Oliver P., Palou A. (2014). Peripheral blood mononuclear cells as a potential source of biomarkers to test the efficacy of weight-loss strategies. Obesity.

[26] Reynés B., Priego T., Cifre M., Oliver P., Palou A. (2018). Peripheral blood cells, a transcriptomic tool in nutrigenomic and obesity studies: Current state of the art. Compr. Rev. Food Sci. Food Saf..

[27] He C.-S., Fraser W.D., Gleeson M. (2014). Influence of vitamin d metabolites on plasma cytokine concentrations in endurance sport athletes and on multiantigen stimulated cytokine production by whole blood and peripheral blood mononuclear cell cultures. ISRN Nutr..

[28] Stepien M., Nugent A.P., Brennan L. (2014). Metabolic profiling of human peripheral blood mononuclear cells: Influence of vitamin d status and gender. Metabolites.

[29] Zheng L., Sun Z., Li J., Zhang R., Zhang X., Liu S., Li J., Xu C., Hu D., Sun Y. (2008). Pulse pressure and mean arterial pressure in relation to ischemic stroke among patients with uncontrolled hypertension in rural areas of China. Stroke.

[30] Friedewald W.T., Levy R.I., Fredrickson D.S. (1972). Estimation of the concentration of low-density lipoprotein cholesterol in plasma, without use of the preparative ultracentrifuge. Clin. Chem..

[31] Cormier H., Rudkowska I., Lemieux S., Couture P., Vohl M. (2016). Expression and sequence variants of inflammatory genes; effects on plasma inflammation biomarkers following a 6-week supplementation with fish oil. Int. J. Mol. Sci..

[32] Gomez-Delgado F., Alcala-Diaz J.F., Garcia-Rios A., Delgado-Lista J., Ortiz-Morales A., Rangel-Zuñiga O., Tinahones F.J., Gonzalez-Guardia L., Malagon M.M., Bellido-Muñoz E. (2014). Polymorphism at the TNF-alpha gene interacts with Mediterranean diet to influence triglyceride metabolism and inflammation status in metabolic syndrome patients: From the CORDIOPREV clinical trial. Mol. Nutr. Food Res..

[33] Rangel-Zúñiga O.A., Corina A., Lucena-Porras B., Cruz-Teno C., Gómez-Delgado F., Jiménez-Lucena R., Alcalá-Díaz J.F., Haro-Mariscal C., Yubero-Serrano E.M., Delgado-Lista J. (2016). TNFA gene variants related to the inflammatory status and its association with cellular aging: From the CORDIOPREV study. Exp. Gerontol..

[34] Keramat L., Sadrzadeh-Yeganeh H., Sotoudeh G., Zamani E., Eshraghian M., Mansoori A., Koohdani F. (2017). Apolipoprotein A2 -265T/C polymorphism interacts with dietary fatty acids intake to modulate inflammation in type 2diabetes mellitus patients. Nutrition.

[35] Koohdani F., Sadrzadeh-Yeganeh H., Djalali M., Eshraghian M., Zamani E., Sotoudeh G., Mansournia M.-A., Keramat L. (2016). APO A2 − 265T/C polymorphism is associated with increased inflammatory responses in patients with type 2 diabetes mellitus. Diabetes Metab. J..

[36] Delgado-Lista J., Perez-Jimenez F., Tanaka T., Perez-Martinez P., Jimenez-Gomez Y., Marin C., Ruano J., Parnell L., Ordovas J.M., Lopez-Miranda J. (2007). An apolipoprotein A-II polymorphism (-265T/C, rs5082) regulates postprandial response to a saturated fat overload in healthy men. J. Nutr..

[37] Corella D., Peloso G., Arnett D.K., Cupples L.A., Tucker K., Lai C., Parnell L.D., Coltell O., Lee Y., Jose M. (2010). APOA2, Dietary fat and body mass index: Replication of a gene-diet interacton in three independent populations. Arch. Intern. Med..

[38] Corella D., Arnett D.K., Tsai M.Y., Kabagambe E.K., Peacock J.M., Hixson J.E., Straka R.J., Province M., Lai C.-Q., Parnell L.D. (2007). The -256T>C polymorphism in the apolipoprotein a-ii gene promoter is associated with body mass index and food intake in the genetics of lipid lowering drugs and diet network study. Clin. Chem..

[39] Zamani E., Sadrzadeh-Yeganeh H., Sotoudeh G., Keramat L., Eshraghian M., Rafiee M., Koohdani F. (2017). The interaction between ApoA2 −265T>C polymorphism and dietary fatty acids intake on oxidative stress in patients with type 2 diabetes mellitus. Eur. J. Nutr..

[40] Sutton A., Imbert A., Igoudjil A., Descatoire V., Cazanave S., Pessayre D., Degoul F. (2005). The manganese superoxide dismutase Ala16Val dimorphism modulates both mitochondrial import and mRNA stability. Pharmacogenet. Genomics.

[41] Bresciani G., Cruz I.B.M., de Paz J.A., Cuevas M.J., González-Gallego J. (2013). The MnSOD Ala16Val SNP: Relevance to human diseases and interaction with environmental factors. Free Radic. Res..

[42] Capeleto D., Barbisan F., Azzolin V., Dornelles E.B., Rogalski F., Teixeira C.F., Machado A.K., Cadoná F.C., da Silva T., Duarte T. (2015). The anti-inflammatory effects of resveratrol on human peripheral blood mononuclear cells are influenced by a superoxide dismutase 2 gene polymorphism. Biogerontology.

[43] Yeh H.-L., Kuo L.-T., Sung F.-C., Yeh C.-C. (2018). Association between Polymorphisms of Antioxidant Gene (MnSOD, CAT, and GPx1) and Risk of Coronary Artery Disease. Biomed Res. Int..

[44] Li J.Y., Tao F., Wu X.X., Tan Y.Z., He L., Lu H. (2015). Polymorphic variations in manganese superoxide dismutase (MnSOD) and endothelial nitric oxide synthase (eNOS) genes contribute to the development of type 2 diabetes mellitus in the Chinese Han population. Genet. Mol. Res..

[45] Paludo F.J.O., Bristot I.J., Alho C.S., Gelain D.P., Moreira J.C.F. (2014). Effects of 47C allele (rs4880) of the SOD2 gene in the production of intracellular reactive species in peripheral blood mononuclear cells with and without lipopolysaccharides induction. Free Radic. Res..

[46] Tian C., Fang S., Du X., Jia C. (2011). Association of the C47T polymorphism in SOD2 with diabetes mellitus and diabetic microvascular complications: A meta-analysis. Diabetologia.

[47] Duarte T., da Cruz I.B.M., Barbisan F., Capelleto D., Moresco R.N., Duarte M.M.M.F. (2016). The effects of rosuvastatin on lipid-lowering, inflammatory, antioxidant and fibrinolytics blood biomarkers are influenced by Val16Ala superoxide dismutase manganese-dependent gene polymorphism. Pharmacogenomics J..

[48] Kakkoura M.G., Demetriou C.A., Loizidou M.A., Loucaides G., Neophytou I., Malas S., Kyriacou K., Hadjisavvas A. (2016). MnSOD and CAT polymorphisms modulate the effect of the Mediterranean diet on breast cancer risk among Greek-Cypriot women. Eur. J. Nutr..

[49] Middelberg R.P.S., Ferreira M.A.R., Henders A.K., Heath A.C., Madden P.A.F., Montgomery G.W., Martin N.G., Whitfield J.B. (2011). Genetic variants in LPL, OASL and TOMM40/APOE-C1-C2-C4 genes are associated with multiple cardiovascular-related traits. BMC Med. Genet..

[50] Johansen C.T., Wang J., Lanktree M.B., Cao H., McIntyre A.D., Ban M.R., Martins R.A., Kennedy B.A., Hassell R.G., Visser M.E. (2010). Excess of rare variants in genes identified by genome-wide association study of hypertriglyceridemia. Nat. Genet..

[51] Chasman D.I., Paré G., Mora S., Hopewell J.C., Peloso G., Clarke R., Cupples L.A., Hamsten A., Kathiresan S., Mälarstig A. (2009). Forty-three loci associated with plasma lipoprotein size, concentration, and cholesterol content in genome-wide analysis. PLoS Genet..

[52] Surakka I., Horikoshi M., Mägi R., Sarin A.-P., Mahajan A., Lagou V., Marullo L., Ferreira T., Miraglio B., Timonen S. (2015). The impact of low-frequency and rare variants on lipid levels. Nat. Genet..

[53] Pollin T.I., Jablonski K.A., McAteer J.B., Saxena R., Kathiresan S., Kahn S.E., Goldberg R.B., Altshuler D., Florez J.C. (2011). Diabetes prevention program research group, for the D. P. P. R. triglyceride response to an intensive lifestyle intervention is enhanced in carriers of the GCKR Pro446Leu polymorphism. J. Clin. Endocrinol. Metab..

[54] Rousseaux J., Duhamel A., Dumont J., Dallongeville J., Molnar D., Widhalm K., Manios Y., Sjöström M., Kafatos A., Breidenassel C. (2015). The n-3 long-chain PUFAs modulate the impact of the *GCKR* Pro446Leu polymorphism on triglycerides in adolescents. J. Lipid Res..

[55] Perez-Martinez P., Delgado-Lista J., Garcia-Rios A., Mc Monagle J., Gulseth H.L., Ordovas J.M., Shaw D.I., Karlström B., Kiec-Wilk B., Blaak E.E. (2011). Glucokinase regulatory protein genetic variant interacts with omega-3 pufa to influence insulin resistance and inflammation in metabolic syndrome. PLoS ONE.

[56] Zimmermann E., Skogstrand K., Hougaard D.M., Astrup A., Hansen T., Pedersen O., Sørensen T.I.A., Jess T. (2011). Influences of the common FTO rs9939609 variant on inflammatory markers throughout a broad range of body mass index. PLoS ONE.

[57] Frayling T.M., Timpson N.J., Weedon M.N., Zeggini E., Freathy R.M., Lindgren C.M., Perry J.R.B., Elliott K.S., Lango H., Rayner N.W. (2007). A Common variant in the FTO gene is associated with body mass index and predisposes to childhood and adult obesity. Science.

[58] Fisher E., Schulze M.B., Stefan N., Häring H.-U., Döring F., Joost H.-G., Al-Hasani H., Boeing H., Pischon T. (2009). Association of the FTO rs9939609 single nucleotide polymorphism with c-reactive protein levels. Obesity.

[59] Phillips C.M., Kesse-Guyot E., McManus R., Hercberg S., Lairon D., Planells R., Roche H.M. (2012). High dietary saturated fat intake accentuates obesity risk associated with the fat mass and obesity-associated gene in adults. J. Nutr..

[60] Gustavsson J., Mehlig K., Leander K., Berg C., Tognon G., Strandhagen E., Björck L., Rosengren A., Lissner L., Nyberg F. (2015). FTO gene variation, macronutrient intake and coronary heart disease risk: A gene-diet interaction analysis. Eur. J. Nutr..

[61] Corella D., Arnett D.K., Tucker K.L., Kabagambe E.K., Tsai M., Parnell L.D., Lai C.-Q., Lee Y.-C., Warodomwichit D., Hopkins P.N. (2011). A high intake of saturated fatty acids strengthens the association between the fat mass and obesity-associated gene and BMI. J. Nutr..

[62] Kumari R., Kumar S., Ahmad M.K., Singh R., Kant Kumar S., Pradhan A., Chandra S., Kumar S. (2018). Promoter variants of TNF-α rs1800629 and IL-10 rs1800871 are independently associated with the susceptibility of coronary artery disease in north Indian. Cytokine.

[63] Ansari W.M., Humphries S.E., Naveed A.K., Khan O.J., Khan D.A. (2017). Influence of cytokine gene polymorphisms on proinflammatory/anti-inflammatory cytokine imbalance in premature coronary artery disease. Postgrad. Med. J..

[64] Kumar P., Misra S., Kumar A., Pandit A.K., Chakravarty K., Prasad K. (2016). Association between tumor necrosis factor-α (-238G/A and -308G/A) gene polymorphisms and risk of ischemic stroke: A meta-analysis. Pulse.

[65] Oki E., Norde M.N., Carioca A.A.F., Souza J.M.P., Castro I.A., Marchioni D.M.L., Fisberg R.M., Rogero M.M. (2017). Polymorphisms of the TNF-α gene interact with plasma fatty acids on inflammatory biomarker profile: A population-based, cross-sectional study in São Paulo, Brazil. Br. J. Nutr..

[66] West M., Greason E., Kolmakova A., Jahangiri A., Asztalos B., Pollin T.I., Rodriguez A. (2009). Scavenger receptor class B type I protein as an independent predictor of high-density lipoprotein cholesterol levels in subjects with hyperalphalipoproteinemia. J. Clin. Endocrinol. Metab..

[67] Perry J.R.B., Voight B.F., Yengo L., Amin N., Dupuis J., Ganser M., Grallert H., Navarro P., Li M., Qi L. (2012). Stratifying type 2 diabetes cases by BMI identifies genetic risk variants in LAMA1 and enrichment for risk variants in lean compared to obese cases. PLoS Genet..

[68] Xiang L., Wu H., Pan A., Patel B., Xiang G., Qi L., Kaplan R.C., Hu F., Wylie-Rosett J., Qi Q. (2016). FTO genotype and weight loss in diet and lifestyle interventions: A systematic review and meta-analysis. Am. J. Clin. Nutr..

[69] Whitworth J.A., World Health Organization, international society of hypertension writing group (2003). 2003 World Health Organization (WHO)/International Society of Hypertension (ISH) statement on management of hypertension. J. Hypertens..

[70] Welty F.K. (2013). How do elevated triglycerides and low HDL-cholesterol affect inflammation and atherothrombosis?. Curr. Cardiol. Rep..

[71] Giannini C., Santoro N., Caprio S., Kim G., Lartaud D., Shaw M., Pierpont B., Weiss R. (2011). The triglyceride-to-HDL cholesterol ratio: Association with insulin resistance in obese youths of different ethnic backgrounds. Diabetes Care.

[72] Kim-Dorner S.-J., Deuster P.A., Zeno S.A., Remaley A.T., Poth M. (2010). Should triglycerides and the triglycerides to high-density lipoprotein cholesterol ratio be used as surrogates for insulin resistance?. Metabolism.

[73] Mostafa S.A., Davies M.J., Morris D.H., Yates T., Srinivasan B.T., Webb D., Brady E., Khunti K. (2012). The association of the triglyceride-to-HDL cholesterol ratio with insulin resistance in white european and south asian men and women. PLoS ONE.

[74] Miralles C.S.W., Wollinger L.M., Marin D., Genro J.P., Contini V., Dal Bosco S.M. (2015). Waist-to-height ratio (WHtR) and triglyceride to HDL-c ratio (TG/HDL-c) as predictors of cardiometabolic risk. Nutr. Hosp..

[75] Jayanthi R., Srinivasan A.R., Hanifah M., Maran A.L. (2017). Associations among Insulin Resistance, Triacylglycerol/High Density Lipoprotein (TAG/HDL ratio) and Thyroid hormone levels—A study on Type 2 diabetes mellitus in obese and overweight subjects. Diabetes Metab. Syndr. Clin. Res. Rev..

[76] Goodarzi M.O. (2018). Genetics of obesity: What genetic association studies have taught us about the biology of obesity and its complications. Lancet Diabetes Endocrinol..

[77] Loos R.J. (2018). The genetics of adiposity. Curr. Opin. Genet. Dev..

[78] Nauroth J.M., Liu Y.C., Van Elswyk M., Bell R., Hall E.B., Chung G., Arterburn L.M. (2010). Docosahexaenoic Acid (DHA) and Docosapentaenoic Acid (DPAn-6) algal oils reduce inflammatory mediators in human peripheral mononuclear cells in vitro and paw edema In Vivo. Lipids.

[79] Walker C., West A., Browning L., Madden J., Gambell J., Jebb S., Calder P. (2015). The pattern of fatty acids displaced by EPA and DHA following 12 months supplementation varies between blood cell and plasma fractions. Nutrients.

[80] Huang C.-W., Chien Y.-S., Chen Y.-J., Ajuwon K.M., Mersmann H.M., Ding S.-T. (2016). Role of n-3 polyunsaturated fatty acids in ameliorating the obesity-induced metabolic syndrome in animal models and humans. Int. J. Mol. Sci..

[81] Marigorta U.M., Gibson G. (2014). A simulation study of gene-by-environment interactions in GWAS implies ample hidden effects. Front. Genet..

[82] Loos R.J.F.F. (2012). Genetic determinants of common obesity and their value in prediction. Best Pract. Res. Clin. Endocrinol. Metab..

[83] de Toro-Martín J., Arsenault B., Després J.-P., Vohl M.-C. (2017). Precision nutrition: A review of personalized nutritional approaches for the prevention and management of metabolic syndrome. Nutrients.

[84] Díaz-Rúa R., Keijer J., Caimari A., van Schothorst E.M., Palou A., Oliver P. (2015). Peripheral blood mononuclear cells as a source to detect markers of homeostatic alterations caused by the intake of diets with an unbalanced macronutrient composition. J. Nutr. Biochem..

[85] Kim M., Song G., Kang M., Yoo H.J., Jeong T., Lee S., Lee J.H. (2016). Replacing carbohydrate with protein and fat in prediabetes or type2 diabetes: Greater effect on metabolites in PBMC than plasma. Nutr. Metab..

[86] Article O. (2018). Effects of n−3 fatty acid supplements in diabetes mellitus. N. Engl. J. Med..

[87] Aung T., Halsey J., Kromhout D., Gerstein H.C., Marchioli R., Tavazzi L., Geleijnse J.M., Rauch B., Ness A., Galan P. (2018). Associations of omega-3 fatty acid supplement use with cardiovascular disease risks meta-analysis of 10 trials involving 77 917 individuals. JAMA Cardiol..

